# Protective Effects of Intravitreal Injection of the Rho-Kinase Inhibitor Y-27632 in a Rodent Model of Nonarteritic Anterior Ischemic Optic Neuropathy (rAION)

**DOI:** 10.1155/2020/1485425

**Published:** 2020-07-07

**Authors:** Zuohuizi Yi, Liao Chen, Xiaoling Wang, Changzheng Chen, Yiqiao Xing

**Affiliations:** ^1^Eye Center, Wuhan University Renmin Hospital, Wuhan, China; ^2^Department of Ultrasound Imaging, Wuhan University Renmin Hospital, Wuhan, China

## Abstract

**Purpose:**

We sought to explore the effects of intravitreal injection of the Rho-kinase inhibitor Y-27632 in a rodent model of nonarteritic anterior ischemic optic neuropathy (rAION).

**Methods:**

The rAION model was established by using laser-induced photoactivation of intravenously administered Rose Bengal in rats. The rats received intravitreal injections of Y-27632 or PBS 1, 3, and 6 days after rAION induction. Optical coherence tomography (OCT) was performed at 2 days and 4 weeks after induction. Visual evoked potential (VEP) was used to evaluate the visual function at 4 weeks. Brn3a immunofluorescence staining of surviving RGCs and apoptosis assays of RGCs were performed at 4 weeks.

**Results:**

Optic nerve head (ONH) width was significantly reduced in the Y-27632 group compared with that in the PBS group at 2 days after induction (*p* < 0.05). At 4 weeks, the P1 amplitude of flash-VEP (FVEP) in the Y-27632 group was significantly higher than that of the PBS group (*p* < 0.05). The RGC densities in the central and midperipheral retinas in the Y-27632 group were significantly higher than those in the PBS group (*p* < 0.05). Furthermore, there was a significant decrease in apoptotic RGCs in the Y-27632 group than in the PBS group (*p* < 0.05).

**Conclusions:**

Intravitreal injection of Y-27632 had neuroprotective effects on ONH edema, RGC survival, and visual function preservation in rAION.

## 1. Introduction

Nonarteritic anterior ischemic optic neuropathy (NAION) is the leading cause of sudden optic nerve-related vision loss in elderly people [1]. NAION is likely initiated by acute transient hypoperfusion or nonperfusion in the vascular networks of the anterior optic nerve head originating from the short posterior ciliary arteries, leading to the development of optic disc edema and localized compartment syndrome [1]. Nevertheless, the process that leads to NAION is complex and multifaceted, and the exact underlying cellular and molecular mechanisms remain poorly understood [2]. Moreover, there is no universally accepted treatment for NAION [1, 2], partly because NAION is not lethal, which makes it extremely difficult to obtain fresh human specimens for research [2]. Fortunately, primate and rodent models of NAION have been established to facilitate exploration of the underlying mechanisms as well as identification of potential therapies [2–5].

In these models, a laser is used to activate an intravascular photoactive dye to produce superoxide radicals, which then causes capillary thrombosis of the anterior optic nerve and secondary apoptosis of retinal ganglion cells (RGCs) [2]. Studies show that observable optic nerve changes in rodent models of AION (rAION) and primate NAION (pNAION) are similar to the clinical changes observed in human NAION, including optic disc edema, electrophysiological changes, and axonal involvement [2–4]. The histopathologic changes observed in the optic nerve and retina in these animal models are also similar to those seen in the few reported human NAION specimens [6]. Accordingly, use of these models can help elucidate the mechanisms underlying this disease and aid in the development of treatment options.

The Rho family of GTPases consists of small GTP-binding proteins, which belong to the Ras-superfamily. Rho proteins, known as “molecular switches,” play key roles in some cellular signal transduction pathways. Rho-associated protein kinase (ROCK) is an important downstream effector of Rho and, as such, has been studied extensively [7]. The RhoA/ROCK pathway is implicated in the pathophysiology of central nervous system (CNS) diseases such as stroke, optic nerve injury, spinal cord injury (SCI), and neurodegenerative diseases [8–11]. In a rat model of focal cerebral infarction, RhoA was activated and upregulated in the lesion area after cerebral infarction [9]. Evidence from animal studies of optic nerve crush (ONC) and glaucoma reveals that the RhoA/ROCK signaling pathway is activated, which results in inhibited axonal outgrowth as well as induction of apoptosis in RGCs [7, 10, 11]. In a rAION model, optic nerve ischemia results in axonal demyelination, which activates RhoA and further leads to axonal regeneration failure [5]. Similarly, another study found that activation of RhoA in rAION reached a peak at day 8, along with progressive loss of RGCs [12].

A recent pilot study revealed that intravitreal injection (IVI) of the ROCK inhibitor fasudil in recent-onset NAION patients led to a statistically significant functional and structural improvement in the majority of patients [13]. The authors speculated that fasudil may play a therapeutic role by increasing the blood flow of the optic disc and directly acting on damaged neurons in NAION patients, but there is a lack of experimental evidence. In addition, a comparative cohort study revealed that the superficial peripapillary retinal vessel density increased significantly with the application of the topical ROCK inhibitor ripasudil in patients with primary open-angle glaucoma and ocular hypertension, as determined by optical coherence tomography angiography [14]. The results suggested that the topical application of ripasudil in glaucoma patients may vasodilate the peripapillary vessels and improve microcirculation in the optic disc. Furthermore, the neuroprotective effects of different ROCK inhibitors in ONC model have also been reported [7, 10, 11].

Y-27632 is one of the classic ROCK inhibitors based on 4-aminopyridine structure, which is widely used in research [15]. It efficiently inhibits both ROCK-I and ROCK-II by competing with ATP for binding to the ROCK catalytic site [15, 16]. Its therapeutic effect has been reported in some neurodegenerative disease animal models in vivo [15, 16]. Based on these studies, we hypothesized that ROCK inhibitors Y-27632 may exert the protective effects in the NAION animal model. In order to test this hypothesis, we evaluated the effects of IVI of Y-27632 in a rAION model.

## 2. Materials and Methods

### 2.1. Animals

All animal protocols were approved by the Institutional Review Board of Renmin Hospital of Wuhan University and were performed in accordance with the ARVO Statement for the Use of Animals in Ophthalmic and Vision Research. Adult male Sprague-Dawley rats (weight 120–150 g) obtained from Animal Experiment Center of Wuhan University (A3 Laboratory) were used in this study. All animals were kept in a laboratory-controlled environment (18–25°C, 12-hr light-dark cycle) and were permitted free access to food and water.

### 2.2. Study Design

Twenty-four rats were randomized into two groups: one group received an intravitreal injection of 2 *μ*L Y-27632 (1.5 mM, Calbiochem, EMD Biosciences, La Jolla, CA, USA) at 1, 3, and 6 days after the induction of rAION, and the other group received a 2 *µ*L phosphate-buffered saline (PBS) intravitreal injection at the same time points. An additional 12 rats received a sham rAION induction in order to serve as the normal control group. For rAION-induced eyes, spectral-domain optical coherence tomography (SD-OCT) (HRA2, Heidelberg Engineering, Heidelberg, Germany) was performed at 2 and 28 days after rAION induction. Flash-VEP (FVEP) was recorded at 28 days after rAION induction. All rats were euthanized after OCT and FVEP were performed at 28 days after induction, and the appropriate tissues were then prepared according to the requirements of the histological experiments.

### 2.3. Induction of rAION Model

The rAION model was established based on methods used in the previous reports [2–5]. Briefly, after general anesthesia and pupil dilation, a transparent contact lens was placed over the cornea of the rat to visualize the retina and optic nerve head (ONH). Then, 1 ml/kg Rose Bengal (RB) (2.5 mM) was administered intravenously through the tail vein. After, the capillaries of the ONH were directly illuminated with a 532 nm green light laser (NIDEK MC-500 Vixi, Nidek Co., Ltd. Gamagori, Japan), 500 *μ*m in size, and 80 mW in power, for twelve 1 second pulses. After laser activation, RB glowed a bright golden color, indicating a successful photodynamic model. Rats in the control group received a sham operation, which included the laser treatment without RB injection.

### 2.4. Intravitreal Injection

The pupils were dilated with 1% tropicamide, and the eyes received topical anesthesia via 0.4% oxybuprocaine hydrochloride eye drops. Before intravitreal injection, the eyes were irrigated with 0.9% sterilized saline and the periorbital skin was sterilized using 5% povidone iodine. Then, under aseptic conditions, a 33-gauge needle attached to a 10 *μ*l Hamilton Gastight syringe (Hamilton 1701RN SYR; Hamilton Co., Hamilton, KS, USA) was used to inject the treatment agents or PBS intravitreally through the pars plana under a dissecting microscope in order to avoid injury to the lens and retina.

### 2.5. OCT Scan

Heidelberg SD-OCT (HRA2, Heidelberg Engineering, Heidelberg, Germany) was used to acquire ONH width and ganglion cell complex (GCC) thickness. We adjusted the parameters and the focal length of the OCT device to obtain clear images of retinal layers. Five scans were performed for each eye, and the highest quality image was used for analysis. Based on the parameters used in the previous studies [17–19], ONH width was defined as the widest gap between the inner nuclear layers (INL) on both sides of the optic disc, and GCC thickness was defined as the total thickness of the retinal nerve fiber layer (RNFL), the ganglion cell layer (GCL), and the inner plexiform layer (IPL). ONH width was obtained using a linear OCT scan through the center of the optic disc, and GCC thickness was measured using a circular OCT scan around the optic disc.

### 2.6. FVEP Recording

The FVEP test was performed as previously described [17], with some modifications. Rats were dark-adapted for approximately 3 h before VEP testing. A visual electrodiagnostic system (Diagnosys LLC, Lowell, MA, USA) was used to record the FVEP waveforms. While the study eye was tested, a light-proof occluding cover was used on the contralateral eye to avoid interference. The silver-needle recording electrode was placed under the skin of the skull midline anterior to the occipital protuberance, the silver-needle reference electrode was placed in the oral cavity, and the silver-needle ground electrode was placed under the skin of the tail. The settings for FVEP were as follows: a single full-field flash stimulus with flash intensity of 3.0 cd/m^2^, flash rate of 1.9 Hz, filtering between 0.1 Hz and 85 Hz, and at least 50 repeated sweeps until waveforms stabilized. The amplitude of the first positive-going wave (*P*1) of FVEP was recorded and analyzed.

### 2.7. Specimen Preparation

After the rats were euthanized, the eyes, with attached ONs, were removed and immediately fixed in 4% paraformaldehyde for 24 h after puncturing at the cornea. A portion of the eyes were embedded in paraffin, and the retinas were serially sectioned longitudinally along the ONs at 5 *μ*m thickness for later use in a TUNEL assay to detect apoptosis. For the remaining eyes, the anterior segments were removed, with the aid of a microscope, to form eyecups. The vitreous body was digested with hyaluronidase (1 : 500, Sigma Chemicals) for 2 h. The retina was gently dissociated from the remaining eyecup and then incised in a Maltese cross pattern to be flat mounted. Immunofluorescent RGC counting was performed on the flat-mounted retina.

### 2.8. In Situ Terminal Deoxynucleotidyl Transferase dUTP Nick End-Labeling (TUNEL) Assay

TUNEL detection was performed to detect apoptotic cells using a Roche TUNEL assay kit (Indianapolis, IN, US), according to the manufacturer's protocol (In Situ Cell Death Detection Kit, POD Protocol). The retina paraffin sections, which contained the retina at 1-2 mm from the ONH, were selected to be observed under a light microscope (BX53, Olympus, Tokyo, Japan). The number of TUNEL-positive cells in the RGC layer was counted in four high-powered fields (HPF, 400× magnification), and three retina sections per eye were averaged.

### 2.9. Immunofluorescence Labeling of RGCs on Flat-Mounted Retinas

Whole flat-mounted retinas were permeabilized in 0.5% Triton X-100 in PBS (PBST) for 15 min and then blocked in 5% bovine serum albumin (BSA) for 1 h. Then, the retinas were incubated in goat-anti-Brn3a primary antibody (1 : 200 dilution; SC-31985; Santa Cruz Biotechnology, Inc., Santa Cruz, CA) overnight at 4 °C in order to label RGCs. Following extensive washing in PBS, the specimens were incubated with Cy3-labeled donkey anti-goat secondary antibody (Jackson ImmunoResearch, West Grove, PA; 1 : 100) for 1 h at room temperature. Finally, the retinas were flat-mounted with the vitreous side up on a glass slide using an antifading mounting medium and then observed and imaged using a fluorescent digital microscope (BX53, Olympus, Tokyo, Japan). The number of RGCs in each specimen was counted using image analysis software (Image Pro-Plus 6.0; Media Cybernetics, Inc., MD, USA). As described previously [20], central and midperipheral RGC numbers were obtained in the area at a distance of 1 or 3 mm from the center of the retina specimen, respectively. Four random areas (one in each quadrant; 200× magnification) in the central retina and four random areas (one in each quadrant; 200× magnification) in the midperipheral retina were selected for analysis, and the average RGC counts per mm^2^ retinal area, known as the RGC density, was calculated.

### 2.10. Statistical Analysis

All statistical analyses were performed using SPSS statistical software (version 24.0; IBM Corp, NY, USA). The Kruskal–Wallis test was used to evaluate the differences between the control and experimental groups. Values are expressed as mean ± standard deviation (SD), and *p* < 0.05 was considered statistically significant.

## 3. Results

### 3.1. Treatment with Y-27632 Reduced ONH Edema in Acute rAION and Preserved GCC Thickness in Chronic rAION

**Figure 1:**
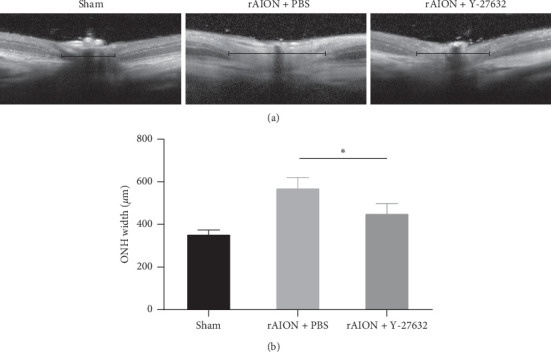
Cross-sectional OCT images of the ONH 2 days after induction. (a) Linear scan across the ONH. The black lines in the images identify the ONH width in each group. (b) Quantification of ONH width. The ONH width in the Y-27632-treated group was significantly reduced compared with the PBS-treated group (454.4 ± 51.2 *μ*m vs. 574.4 ± 53.6 *μ*m, *p* < 0.05, *n* = 12 per group). The asterisk indicates *p* < 0.05, as determined by the Kruskal–Wallis test.

Two days after rAION induction, SD-OCT revealed that the ONH width in the sham, PBS-treated, and Y-27632-treated groups were 357.2 ± 24.1 *μ*m, 574.4 ± 53.6 *μ*m, and 454.4 ± 51.2 *μ*m, respectively. ONH edema, as shown by the ONH width, was significantly higher in both treatment groups compared with that in the sham group (*p* < 0.05). Compared with the PBS-treated group, Y-27632 treatment significantly reduced ONH edema in the acute phase after induction (*p* < 0.05) (Figure 1).

**Figure 2:**
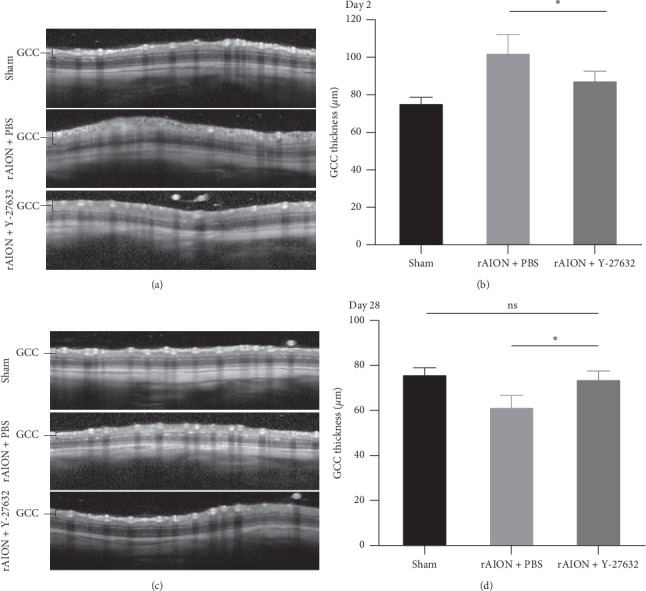
Cross-sectional OCT images displaying GCC thickness at 2 days and 28 days after induction. (a) Circular scan around the optic disc showing GCC thickness at 2 days after induction. (b) Quantification of GCC thickness at 2 days after induction. The mean GCC thickness in the Y-27632-treated group was significantly lower than that of the PBS-treated group at day 2 (*n* = 12 per group). The asterisk indicates *p* < 0.05, as determined by the Kruskal–Wallis test. (c) Circular scan around the optic disc showing GCC thickness at 28 days after induction. (d) Quantification of GCC thickness at 28 days after induction. The mean GCC thickness in the Y-27632-treated group was significantly higher than that of the PBS-treated group after 28 days but was not significantly different from that of the sham group (*n* = 12 per group). The asterisk indicates *p* < 0.05, as determined by the Kruskal–Wallis test.

The mean GCC thickness at day 2 was 75.8 ± 3.7 *μ*m, 102.6 ± 10.5 *μ*m, and 87.9 ± 5.5 *μ*m in the sham, PBS-treated, and Y-27632-treated groups, respectively. The mean GCC thickness in the Y-27632-treated group was lower than that in the PBS-treated group at day 2 (*p* < 0.05) but was higher than that in the sham group (*p* < 0.05). The mean GCC thickness at day 28 was 76.2 ± 3.3 *μ*m, 61.7 ± 5.6 *μ*m, and 74.1 ± 3.9 *μ*m in the sham, PBS-treated, and Y-27632-treated groups, respectively. The mean GCC thickness in the Y-27632-treated group was higher than that in the PBS-treated group at day 28 (*p* < 0.05), which did not differ from the sham group (*p*=0.54) (Figure 2). Thus, Y-27632 treatment can reduce GCC thickness in the acute phase of rAION and preserve GCC thickness in the chronic phase of rAION.

### 3.2. Treatment with Y-27632 Improved *P*1 Amplitudes after rAION Induction

**Figure 3:**
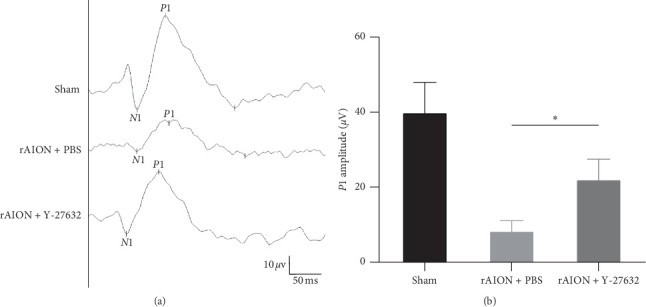
Evaluation of the recovery of damaged optic nerves through FVEP recording at 28 days after induction. (a) Representative FVEP wave after rAION for each group; (b) quantification of *P*1 wave amplitude. The amplitude of the *P*1 wave in the Y-27632-treated group was significantly higher than that in the PBS-treated group (22.2 ± 5.7 *μ*V vs. 8.4 ± 3.1 *μ*V, *p* < 0.05, *n* = 12 per group). The asterisk indicates *p* < 0.05, as determined by the Kruskal–Wallis test.

FVEP analysis performed 28 days after induction showed that the mean amplitudes of the P1 waves in the sham, PBS-treated, and Y-27632-treated groups was 40.0 ± 8.3 *μ*V, 8.4 ± 3.1 *μ*V, and 22.2 ± 5.7 *μ*V, respectively. The amplitudes of the P1 waves in the Y-27632-treated group were significantly higher than those in the PBS-treated group (*p* < 0.05) but lower than those in the sham group (*p* < 0.05) (Figure 3).

### 3.3. Treatment with Y-27632 Reduced RGC Apoptosis after rAION Induction

**Figure 4:**
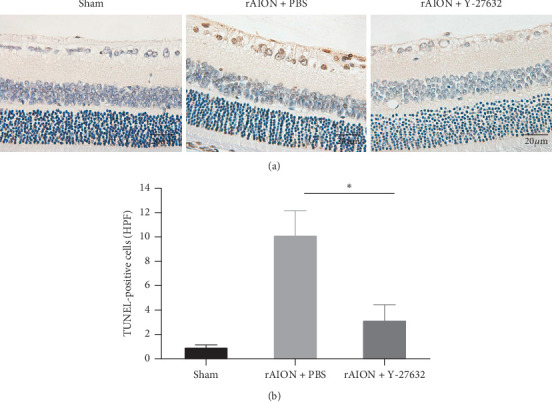
Analysis of RGC apoptosis at 28 days after induction as determined by TUNEL assay. (a) Representative TUNEL images showing apoptotic RGCs in brown; (b) quantification of the number of apoptotic RGCs in each group. The amount of apoptotic RGCs in the Y-27632-treated group was significantly reduced compared with that in the PBS-treated group (*n* = 6 per group). The asterisk indicates *p* < 0.05, as determined by the Kruskal–Wallis test. Scale bar: 20 *μ*m.

The mean number of TUNEL-positive cells in the RGC layer at 28 days after induction was 1.0 ± 0.3 HPF, 10.2 ± 2.1 HPF, and 3.2 ± 1.3 HPF in the sham, PBS-treated, and Y-27632-treated groups, respectively. The amount of apoptotic RGCs in the Y-27632-treated group was significantly lower than the amount in the PBS-treated group (*p* < 0.05) (Figure 4).

### 3.4. Treatment with Y-27632 Preserved RGC Survival following rAION Induction

**Figure 5:**
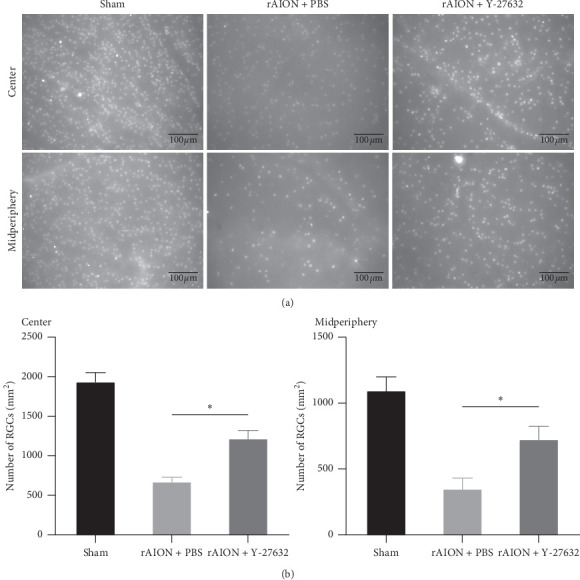
Survival of RGCs at 28 days after induction. (a) Representative central and midperipheral flat-mounted retinas showing RGCs labeled by Brn3a antibody; (b) quantification of RGC densities in the central and midperipheral retinas in each group. The RGC densities within the central and midperipheral retinas were significantly higher in the Y-27632-treated group than in the PBS-treated group (*n* = 6 per group). The asterisk indicates *p* < 0.05, as determined by the Kruskal–Wallis test. Scale bar: 100 *μ*m.

At 28 days after induction, the mean RGC densities within the central retina in the sham, PBS-treated, and Y-27632-treated groups were 1940 ± 121 mm^2^, 676 ± 62 mm^2^, and 1221 ± 109 mm^2^, respectively. The mean RGC densities within the midperipheral retina in the sham, PBS-treated, and Y-27632-treated groups were 1097 ± 111 mm^2^, 352 ± 87 mm^2^, and 727 ± 106 mm^2^, respectively. Treatment with Y-27632 preserved a significantly higher degree of RGC survival in both the central and midperipheral retinas compared with treatment with PBS (*p* < 0.05) (Figure 5).

## 4. Discussion

Our findings in this study demonstrated for the first time that intravitreal injection of the ROCK inhibitor Y-27632 can effectively exert neuroprotective effects in rAION, as evidenced by FVEP analysis and the preservation of RGCs. Our TUNEL assay results showed that Y-27632 treatment reduced RGC apoptosis following rAION induction, which was consistent with our observations of RGCs morphometry in flat-mounted retinas. Furthermore, our OCT findings indicated that the administration of Y-27632 reduced ONH edema during the acute phase and preserved GCC thickness in the chronic phase.

As reported in the previous studies [2, 5], axon and myelin damage could be found in ON ischemia lesions in rAION models. Myelin damage can generate and release myelin-related inhibitory factors, which bind to the axonal membrane protein complex LINGO-1, thereby activating intraneural RhoA. Activated RhoA then activates its downstream effector ROCK, leading to inhibition of actin polymerization and ultimately causing axonal growth cone collapse and axon regeneration failure [5]. In the previous studies of ONC and stroke, researchers found that inhibition of the RhoA/ROCK pathway had neuroprotective effects [8, 10, 11]. Furthermore, RhoA activation was indeed upregulated in the rAION model [5, 12]. Therefore, we sought to determine whether the use of the ROCK inhibitor to reduce active RhoA and ROCK expression may have neuroprotective effects in rAION.

Compared with the systemic administration, intravitreal administration may reduce the potential for systemic effects and increase the drug concentration at the local lesion in the eye [17]. We chose to perform three repeated injections based on information from previous studies. Fard et al. [12] found that RhoA activation peaked 8 days after rAION induction, but there were fewer time points (1, 8, and 14 days) in their study, and a time point between 1–7 days was lacking. Moreover, in local cerebral infarction, RhoA expression in ischemic brain tissue began to increase at 6 h after infarction and peaked at 24 h [9], which was much earlier than what was observed by Fard et al. It is known that the ON is part of the white matter of the brain, so it is reasonable to hypothesize that this early expression pattern of RhoA in ON infarction is similar to that in cerebral infarction. Additionally, repeated injections of ROCK inhibitors were used in several ONC studies [7, 21, 22], and so we also chose repeated intravitreal injections at 1, 3, and 6 days after induction in our study.

Upregulation of the RhoA/ROCK pathway is common in many cardiovascular diseases [23]. ROCK activation can inhibit endothelial nitric oxide synthase (eNOS) and phosphatidylinositol-3-kinase (PI3K)/Akt pathways and reduce nitric oxide (NO) release from endothelial cells [23, 24]. NO is a powerful vasodilator, which can eliminate superoxide radicals and inhibit platelet aggregation and leukocyte adhesion, thus contributing to the repair of ischemic optic nerve damage [25]. Furthermore, activated ROCK causes phosphorylation of its downstream substrate myosin light-chain phosphatase (MLCP), thereby enhancing the contraction of vascular smooth muscle cells [23, 24]. It has been previously reported that ROCK inhibitors could regulate blood flow in many cardiovascular diseases and cerebral vascular diseases via direct effects on vascular smooth muscle or through indirect positive effects on eNOS expression [23]. Sugiyama et al. [24] found that topical or systemic use of the ROCK inhibitor fasudil was able to increase the blood flow of impaired ONH. In our study, Y-27632 treatment reduced ONH edema in the acute phase of rAION, which may be related to this potential role for ROCK inhibitors in improving ONH blood supply.

Apoptosis of RGCs in the rAION model started at 7 days post-induction and lasted for 3-4 weeks following induction [2, 4, 18]. Our study showed that treatment with Y-27632 in rAION markedly rescued RGCs from undergoing apoptosis and eventually preserved RGCs survival. Consistent with our results, previous studies in ONC models have also found that ROCK inhibitors can promote axonal regeneration in RGCs and increase RGCs survival [10, 11]. Yamamoto et al. [10] found that treatment with a ROCK inhibitor promoted RGC survival in ONC models by suppressing oxidative stress pathways. However, the mechanism of RGC apoptosis may be multifaceted, and therefore the antiapoptotic mechanisms involved in Y-27632 treatment in rAION require further analysis. GCC thickness was considered to be a suitable index for analyzing rAION [19]. It was previously reported that the GCC layer experienced significant edema and thickening 1 day after rAION induction, followed by gradual thinning over weeks [19]. The preservation of GCC thickness in the chronic phase of rAION in our study may be related to the ability of Y-27632 to rescue RGCs.

A common feature in rAION models and human NAION is a decrease in amplitude in FVEP analysis [2, 3]. However, a prolonged latency after rAION induction has also been reported [20, 26]. This difference may be related to the severity of ON ischemic infarction in rAION and also suggests that FVEP has a certain degree of variability [20, 26]. Nevertheless, in this study, we found that, in terms of P1 amplitude of FVEP, Y-27632 treatment effectively preserved electrophysiologic visual function after induction.

Although our results demonstrated that intravitreal injection of Y-27632 provides neuroprotective effects in rAION, there are several remaining questions to answer. First, the time points and the doses of Y-27632 treatment in our study were set based on the previous studies [11, 21, 22], but a possible dose-dependent effect for Y-27632 has been reported previously [22], so the optimal dosage and timing should be investigated further. Additionally, in order to better understand the mechanism underlying the therapeutic effect of ROCK inhibitors in rAION, further analysis of the expression of upstream and downstream factors in the activated RhoA/ROCK pathway in rAION is required. Finally, rAION modeling involves a simple photodynamic thrombosis, which is different from the complicated human NAION pathogenesis, making further studies warranted prior to clinical application.

## Data Availability

The data supporting the results of the current article are available from the corresponding author upon request.
